# Validation and Opportunities of Electrocardiographic Imaging: From Technical Achievements to Clinical Applications

**DOI:** 10.3389/fphys.2018.01305

**Published:** 2018-09-20

**Authors:** Matthijs Cluitmans, Dana H. Brooks, Rob MacLeod, Olaf Dössel, María S. Guillem, Peter M. van Dam, Jana Svehlikova, Bin He, John Sapp, Linwei Wang, Laura Bear

**Affiliations:** ^1^Department of Cardiology, Cardiovascular Research Institute Maastricht Maastricht University, Maastricht, Netherlands; ^2^Department of Electrical and Computer Engineering, Northeastern University, Boston, MA, United States; ^3^Biomedical Engineering Department, Scientific Computing and Imaging Institute (SCI), and Cardiovascular Research and Training Institute (CVRTI), The University of Utah, Salt Lake City, UT, United States; ^4^Karlsruhe Institute of Technology, Karlsruhe, Germany; ^5^ITACA, Universitat Politècnican de València, Valencia, Spain; ^6^Donders Institute for Brain, Cognition and Behaviour, Radboud University Nijmegen Medical Center, Nijmegen, Netherlands; ^7^Institute of Measurement Science, Slovak Academy of Sciences, Bratislava, Slovakia; ^8^Department of Biomedical Engineering Carnegie Mellon University, Pittsburgh, PA, United States; ^9^QEII Health Sciences Centre and Department of Medicine, Dalhousie University, Halifax, NS, Canada; ^10^Rochester Institute of Technology, Rochester, NY, United States; ^11^IHU LIRYC, Fondation Bordeaux Université, Inserm U1045 and Université de Bordeaux, Bordeaux, France

**Keywords:** ECG imaging, validation, electrocardiography, electrophysiology, experiment

## Abstract

Electrocardiographic imaging (ECGI) reconstructs the electrical activity of the heart from a dense array of body-surface electrocardiograms and a patient-specific heart-torso geometry. Depending on how it is formulated, ECGI allows the reconstruction of the activation and recovery sequence of the heart, the origin of premature beats or tachycardia, the anchors/hotspots of re-entrant arrhythmias and other electrophysiological quantities of interest. Importantly, these quantities are directly and non-invasively reconstructed in a digitized model of the patient’s three-dimensional heart, which has led to clinical interest in ECGI’s ability to personalize diagnosis and guide therapy. Despite considerable development over the last decades, validation of ECGI is challenging. Firstly, results depend considerably on implementation choices, which are necessary to deal with ECGI’s ill-posed character. Secondly, it is challenging to obtain (invasive) ground truth data of high quality. In this review, we discuss the current status of ECGI validation as well as the major challenges remaining for complete adoption of ECGI in clinical practice. Specifically, showing clinical benefit is essential for the adoption of ECGI. Such benefit may lie in patient outcome improvement, workflow improvement, or cost reduction. Future studies should focus on these aspects to achieve broad adoption of ECGI, but only after the technical challenges have been solved for that specific application/pathology. We propose ‘best’ practices for technical validation and highlight collaborative efforts recently organized in this field. Continued interaction between engineers, basic scientists, and physicians remains essential to find a hybrid between technical achievements, pathological mechanisms insights, and clinical benefit, to evolve this powerful technique toward a useful role in clinical practice.

## Introduction

Electrocardiographic imaging (ECGI) reconstructs the electrical activity of the heart from a dense array of body-surface electrocardiograms and a patient-specific heart-torso geometry. Depending on how the problem is formulated, ECGI allows the reconstruction of the activation and recovery sequence of the heart, the origin of premature beats or tachycardia, the anchors/hotspots of re-entrant arrhythmias and other electrophysiological quantities of interest. Importantly, these quantities are directly and non-invasively reconstructed in a digitized model of the patient’s three-dimensional heart, which allows personalized diagnosis and localized therapy guidance.

Over the past four decades, ECGI has seen considerable development, from purely analytical studies (Rudy et al., 1979; Figuera et al., 2016; Svehlikova et al., 2018), to torso tank (Oster et al., 1997, 1998; Ramanathan and Rudy, 2001; Shome and Macleod, 2007; Bear et al., 2018a) and large animal models (Liu et al., 2012; Oosterhoff et al., 2016; Cluitmans et al., 2017; Bear et al., 2018b) and finally application in humans (Ghanem et al., 2005; Horáček et al., 2011; Haissaguerre et al., 2014; Schulze, 2015; Punshchykova et al., 2016). It is now increasingly used for academic research and in clinical practice. Despite this progress, validation of ECGI remains a significant challenge.

Mathematically, ECGI solves the inverse problem of electrocardiography (i.e., to determine the cardiac electrical source for a given body-surface potential distribution), a problem fundamentally hindered by the fact that a multitude of patterns of cardiac electrical activity can produce similar body-surface potentials. The majority of these “inverse solutions” are physically and physiologically unlikely; therefore, additional constraints are imposed to stabilize the problem and select a more realistic solution, a process that is called “regularization.” Regularization that is based on physical and physiological *a priori* information significantly improves the solvability and robustness of inverse solutions from ECG recordings. ECGI is thus strongly dependent on implementation choices, such as the cardiac source model and the method of regularization. Both these components need to be taken into account by the validation approach, which is not always straightforward. A second aspect that makes ECGI validation challenging is the difficulty in obtaining highly detailed and localized invasive ground-truth data in *in vivo* animals or humans. Finally, ECGI is strongly dependent on the specific clinical application of interest, as each application will yield a need for different quantitative parameters and their validation.

The aims of this paper are:

(1) To review the current status of validation of ECGI, as recent overviews on this topic are lacking (Sections Forms of Validation, Cardiac Source Models, Technical Validation, Pathological Validation, and Clinical (and Socioeconomic) Validation);
(2) To highlight which challenges in ECGI validation remain to facilitate clinical adoption; this includes commercial and socioeconomic challenges (Section Clinical (and Socioeconomic) Validation);
(3) To provide a consensus on the “best” ways to perform ECGI validation in the future (Section Consensus on Designing a Validation Study).

## Forms of Validation

**FIGURE 1:**
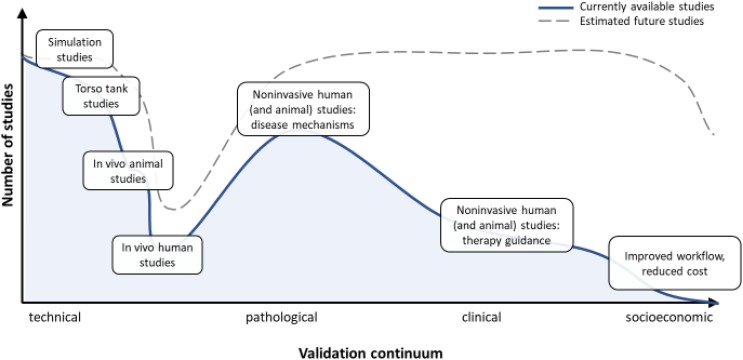
Describes the validation continuum, from purely technical studies to pathological/clinical validation and socioeconomic benefit studies. Whereas the technical validation of ECGI is extensive, as is its use in disease mechanisms studies and validation of the true clinical benefit is still lacking.

Given the increased clinical use of ECGI over the recent past, there is an associated need for ECGI validation. Validation studies come in three different forms: technical, pathological, or clinical validation (see **Figure 1**).

The first form evaluates the **technical** accuracy and performance of ECGI for reconstructing the value of specific electrophysiological quantities. The relevant quantities include transmembrane voltage, electrograms, activation and recovery times, and related features that can be quantified. The accuracy of these features is not only influenced by the inverse or regularization methods that are used, but also by the pre-processing (e.g., techniques that filter or average the recorded body-surface potentials or improve the geometrical accuracy, methods to deal with poor quality signals, etc.) and post-processing methods that are used to extract features that are not directly available from inverse solutions (e.g., the calculation of activation time or other temporal fiducials from a reconstructed electrogram, or the use of phase mapping for rotor detection). Technical validation studies seek to evaluate these quantifiable features, generally irrespective of the underlying disease or clinical setting.

The second category of ECGI validation studies is to define the **pathological** accuracy and performance of ECGI, i.e., its capability to extract features applicable to a specific pathology or arrhythmia. For example, determining the origin of a premature ventricular complex (PVC) requires a different approach than determining the fractionation of local electrograms in myocardial infarction, or determining the region of large repolarization gradients in patients susceptible to ventricular fibrillation (VF). For each of these types of validation, the exact value of the reconstructed quantities may not be as important as their ability to separate healthy from diseased states. For example, it may matter less whether ECGI can determine the exact value of the steepness of a repolarization gradient (which, for example, may depend on mesh coarseness) as long as it can reliably differentiate a proarrhythmic gradient from a normal gradient (Vijayakumar et al., 2014).

The third category of validation studies evaluates the **clinical** accuracy and performance of ECGI, i.e., its benefit in daily clinical practice. The main focus of these studies is not the accuracy of reconstructed or post-processed parameters, but their influence on clinical decision making. For example, ECGI might provide improved therapy outcomes in atrial fibrillation (AF) ablation, improved workflow in the cardiac electrophysiology laboratory, reduced radiation burden or procedural times, etc.

Although we divide validation into three distinct categories, there is often overlap between them, particularly in the case of “pathological” and “clinical” validation. Here we used these categories to emphasize the difference between using ECGI to investigate disease mechanisms (in which validation may focus on parameters directly reconstructed by ECGI) and using ECGI for clinical applications (in which validation may focus on indirect parameters such as patient outcome and reduced procedure costs).

Each validation form has different requirements in terms of data, metrics and analysis (**Figure 1**). In the remainder of this review, we assess the current status of each validation form, discuss the different issues surrounding validation, and provide a consensus as to the best approach to overcome these challenges to arrive at clinically relevant studies and applications.

## Cardiac Source Models

Validating an ECGI formulation begins by defining the cardiac source to be reconstructed. Many of the original ECGI formulations approximated the cardiac source as either a single or multiple equivalent dipoles (Bayley and Berry, 1962; Rudy and Messinger-Rapport, 1988) or equivalent dipole layer (van Oosterom, 2004). Despite these source models being “equivalent” to the “ground truth” cardiac electrical activity they represent (in the sense that the models each fully represent cardiac electrical activity from a biophysical standpoint), there are no obvious physiological links, making experimental and/or clinical validation difficult.

**FIGURE 2:**
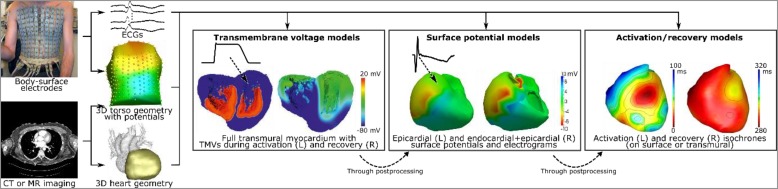
Electrocardiographic imaging requires recording of body-surface potentials and the acquisition of a torso-heart geometry through computed tomography (CT) or magnetic resonance (MR) imaging. The electrical characteristics of the heart can then be computed by employing one of the “source models,” i.e., models of cardiac electrical activity that explain the recorded body-surface potentials. Globally, these source models are categorized as: transmembrane voltage (TMV) models, surface-potential models (either epicardial-only or endo-epicardial surface), and activation/recovery-based models. It is possible to compute surface potentials and electrograms from TMVs, and to compute activation and recovery sequences from electrograms, but not the other way around. Image in part reproduced with permission from: Cluitmans (2016); Weiss et al. (2007), and Van Dam et al. (2009).

**Figure 2** illustrates the three current predominant cardiac source models and the features that can be derived from them: transmembrane voltage-based models, extracellular-potential based models, and activation/recovery-based models. The definition of an appropriate ground truth for comparison among these cardiac source models can be difficult, particularly when using experimental or clinical data sets. Often, investigators need to be satisfied with a derived ground truth data (e.g., activation times) or a ground truth hampered by error due to experimental limitations.

### Transmembrane Voltage-Based Model

Taking the transmembrane voltage (TMV, i.e., the potential difference across the cell membrane of a cardiomyocyte, or a continuum approximation of that quantity), as a cardiac source model is the closest approximation to the true cardiac electrical source. Transmural reconstructions of TMV are possible (He et al., 2003; Wang et al., 2010; Potyagaylo et al., 2012), but a reformulation from Geselowitz (assuming equal anisotropy ratios of intracellular and extracellular conductivity tensors) also allows for a reconstruction of TMV on the endo- and epicardial surfaces (Simms and Geselowitz, 1995).

Reconstructions of TMV support identification of depolarized and repolarized regions in the heart and their temporal evolution. They also have the ability to outline areas of reduced amplitude, or completely absent TMV, e.g., ischemic, fibrotic, or border-zone areas. However, TMVs cannot be measured directly in the clinical environment, and thus any validation has to occur indirectly via the extracellular signals that TMV distributions create. Extracellular potentials on the endocardial and epicardial surfaces and activation and recovery times can easily be calculated from TMV distributions and then measured in experiments.

Impressed currents (i.e., the gradient of the TMV multiplied by the intracellular conductivity) can also be seen as sources of electrophysiological signals. In the original formulation, the impressed currents are vector fields, thus increasing the number of unknown properties by a factor of three. Thus, some studies propose to reconstruct only the normal or the tangential component of the impressed currents (Grace et al., 2017). For the case of equal anisotropy ratios of conductivity, the impressed currents can also be replaced by an equivalent dipole layer with equivalent current density distributions orthogonal to the surface of the heart (Janssen et al., 2017) (also see the EDL model in Section Activation/Recovery-Based Models).

An advantage of TMV or impressed current based solutions is that *a priori* physiological knowledge about the action potential propagation, available through various mathematical models, can be used to constrain the reconstruction. The challenge in terms of validation, on the other hand, is that TMVs or impressed currents are difficult to obtain experimentally or clinically.

### Extracellular Potential-Based Model

Potential-based models represent the cardiac source using extracellular potentials on the heart surface. There are several possible definitions of this cardiac surface, typically including only the ventricular or atrial cavities. The most common definition uses the epicardial surface, often artificially closed across the valves and the base. The surface can also be defined as both the endocardium and epicardium, leaving an opening at the valves. Both versions are presented for the ventricles in **Figure 2**. The key requirement for most methods is that the surface encompasses all active electrical sources.

Two key features are directly available from a potential-based model: electrograms and potential maps. Cardiac electrograms are the change in potential over time at a single point on the heart, and potential maps show the potential distribution over the surface at a specific time point. One benefit of potential-based models in terms of validation is that these features are much more easily measurable than TMVs and can be recorded during experimental or catheter-based procedures, even in humans. Moreover, like TMVs, additional information contained within the electrograms, such as activation and recovery processes, can be extracted through post-processing. On the other hand, while potential-based models can provide information on both epicardial and endocardial surfaces (Rudy and Burnes, 1999), direct reconstruction of transmural propagation is currently not possible. Importantly, many potential-based implementations of ECGI only provide epicardial reconstructions, without endocardial or septal potentials. In general, the extracellular approach (formulated either just epicardial or endocardial and epicardial) is the basis of some commercial systems and as such may currently have the highest clinical relevance of all source formulations.

### Activation/Recovery-Based Models

The purpose of activation/recovery-time based models is to obtain the local times of activation or recovery directly, without reconstruction of TMVs or extracellular potentials as intermediate. As such they provide a highly stylized, simplified sparse parameterization that captures key aspects of the underlying biophysics. Activation times are described as the time of arrival of the depolarization phase of an action potential. Similarly, recovery times capture the timing of the repolarization phase. Both quantities can be defined either through the 3-dimensional (3D) myocardial wall (Han et al., 2011), or on the heart surface, including both the epicardium and endocardium (Erem et al., 2014). Examples of such methods include the equivalent double layer (EDL) model and the 3D cardiac electrical imaging (3DCEI) model:

• Equivalent Double Layer Model (EDL)
   The macroscopic EDL model (Van Oosterom, 2001) represents the entire electrical activity of the atria or ventricles at any time instant (van Oosterom and Jacquemet, 2005a). This source model stems from the classic bioelectrical double layer as an equivalent source of the currents generated at the boundary between active and resting cells during depolarization, described by Wilson et al. (1933). Initially, this current dipole layer model was used to describe the activity at the front of a depolarization wave propagating through the myocardium. Later, Salu expressed the equivalence between the double layer at the wave front and a uniform double layer at the depolarized part of the surface bounding the myocardium (Salu, 1978), based on solid angle theory (van Oosterom, 2002) (see **Figure 2**). Later, Geselowitz showed, using a bidomain model, that the actual *current* source distribution within the heart is equivalent to a double layer at the surface of the myocardium with a strength proportional to the local TMV (Geselowitz, 1989, 1992; van Oosterom and Jacquemet, 2005b). The waveform of the TMV at each location on the myocardial surface (both endocardium and epicardium) is described by at least two parameters: the local activation and recovery times. Consequently, the source parameters of the EDL model also consist of the activation and recovery times. More complex versions allow as many as seven parameters (van Oosterom, 2004). One challenge of this model is that the relationship between the source parameters and the source strength is non-linear.
• 3D Cardiac Electrical Imaging (3DCEI)
   While the EDL model allows estimating activation or recovery times over the heart surface (including both epicardium and endocardium), the 3DCEI model aims to reconstruct cardiac activation and recovery process throughout the entire 3D myocardium – including epicardium, endocardium, and intramural tissues (MacLeod et al., 2000; He et al., 2003). While 3DCEI is applicable to both ventricles and atria, its main application is for imaging the ventricular activation sequence (Han et al., 2011) from the inversely reconstructed equivalent current densities (He and Wu, 2001), or imaging the ventricular repolarization process through reconstruction of the activation-repolarization interval at each point within the 3D ventricles (Wang et al., 2013). The relationship between cardiac equivalent current density and local activation time is established based on the “peak criteria” reflecting the fundamental biophysics of cardiac activation (MacLeod et al., 2000). This model has been evaluated extensively in *in vivo* animal models with simultaneous 3D intracardiac mapping in rabbits (Han et al., 2011; Duchateau et al., 2016) and in dogs (Cluitmans et al., 2017), as well in scarred myocardial tissue (Wang et al., 2013).

To solve the inverse problem using these source models usually requires an initial estimate of the activation and recovery times, which is then improved through numerical iteration. An advantage of these approaches is that *a priori* physiological knowledge can be used to obtain very reasonable initial activation and recovery times (Van Dam et al., 2009), thus reducing convergence time and improving accuracy. At the same time, it remains unclear how well the models perform when assumptions of the underlying physiology are violated, e.g., when scar tissue is present but is not incorporated in the model (Erem et al., 2014). For normal healthy subjects and patients with idiopathic PVC, this method has been shown to work well (Van Dam et al., 2009). Current research focuses on developing initial estimate algorithms that deal with inhomogeneous cardiac activation, e.g., when scar tissue is present.

## Technical Validation

Technical validation of ECGI is the most quantitative validation approach, explicitly comparing the ECGI reconstruction to ground truth to determine its absolute accuracy irrespective of the underlying pathology. Technical validation encompasses a broad range of cardiac source models, forward model formulations, inverse methods, and pre- and post-processing techniques. These studies are typically executed using either analytical (Rudy et al., 1979) or simulated potentials (Dubois et al., 2016; Figuera et al., 2016; Svehlikova et al., 2018), *ex vivo* torso tank experiments (Oster et al., 1997; Shome and Macleod, 2007; Bear et al., 2018a) and *in vivo* animal studies (Liu et al., 2012; Oosterhoff et al., 2016; Cluitmans et al., 2017; Bear et al., 2018b), with more limited results from humans (Ghanem et al., 2005; Sapp et al., 2012; Erem et al., 2014; Schulze, 2015; Punshchykova et al., 2016). For simplicity, here we have organized technical validation according to the different features that may be extracted from ECGI, irrespective of source models. These features provide scientists and clinicians with information about cardiac electrical activity and can either be provided directly by the cardiac source model chosen (i.e., electrograms when using a potential-based approach) or extracted through further post-processing of the ECGI-reconstructed signals (i.e., activation or phase mapping from electrograms), as summarized in **Figure 2**.

### Transmembrane Voltages

While validation of reconstructed TMVs is possible using simulated data, it is nearly impossible experimentally and clinically as the ground truth data can only be obtained through optical mapping, monophasic action potentials recording, or patch clamping. These are all techniques that are currently difficult to obtain *in vivo* and the latter two are infeasible over more than a very few sites in a whole heart. If these data are available, they serve as the optimal approach for comparing magnitude and shape of different phases, particularly depolarization and repolarization. If these data are not available, the focus of validation shifts to post-processed values of TMPs, such as unipolar electrogram morphology, activation and recovery isochrones, etc.

### Electrograms

**FIGURE 3:**
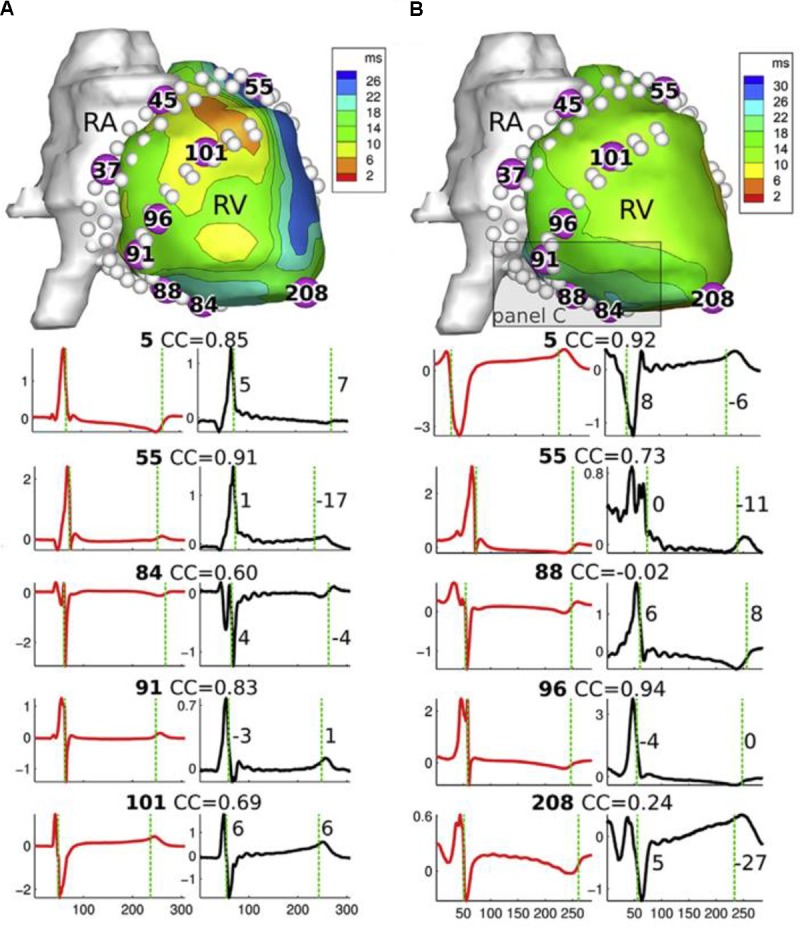
Top: epicardial geometry with reconstructed potentials from a canine *in vivo* study, for a sinus beat **(A)** and a LV-paced beat **(B)**. White dots indicate the position of invasive electrodes used for validation recordings. Bottom: recorded (red) and ECGI-reconstructed (black) electrograms for electrodes numbered in geometry above. CC, correlation coefficient between recorded and reconstructed electrograms. RA, right atrijm; RV, right ventricle. Adapted with permission from Cluitmans et al. (2017).

Electrograms are one of the most common features reconstructed with ECGI, as they provide useful information to clinicians, directly relatable to invasive recordings. Ground truth data is available through simulations (Simms and Geselowitz, 1995; Wang et al., 2010; Figuera et al., 2016; Janssen et al., 2017), recordings obtained with epicardial, endocardial and transmural electrode arrays (with upwards of 200 electrodes) in *ex vivo* (Oster et al., 1997; Shome and Macleod, 2007; Bear et al., 2018b) and *in vivo* (Zhang et al., 2005; Han et al., 2011; Liu et al., 2012; Oosterhoff et al., 2016; Cluitmans et al., 2017; Bear et al., 2018b) experimental models, and invasive mapping clinically (Ghanem et al., 2005; Sapp et al., 2012; Punshchykova et al., 2016). Most validation studies to date use a global evaluation of the QRS, T, or QRS-T waveform reconstruction using correlation and/or error metrics, to demonstrate the accuracy in the overall topology and/or amplitude of electrograms (example in **Figure 3**). Persistent challenges include assessing spatial accuracy (Cluitmans et al., 2017; Bear et al., 2018b) and the sensitivity of results to different sources of error, i.e., cardiac motion (MacLeod et al., 2000; Cluitmans et al., 2017). To date, while qualitative assessment of reconstructed electrograms is often described, comprehensive quantification of the presence/absence of detailed characteristics in reconstructed electrograms has never been performed, including fractionation, low/high amplitudes, epicardial/endocardial source, ST-segment elevation, etc. The inaccurate reconstruction of these characteristics is often missed using a global evaluation. Metrics to quantify the presence of such local features are needed as this information can be critical to the diagnosis and/or treatment of particular pathologies (see Section Metrics for Validation).

Unipolar electrograms on the cardiac surface are influenced by what are known as “far-field” effects from electrical activity in other parts of the myocardium, this influence varying with electrode size and filtering techniques. Since ECGI electrogram reconstructions may be even more sensitive to such effects given the ill-posedness of the inverse problem, care is required in interpreting validation results, especially if the surface model does not include the endocardium or if validation measurements are only available on either the endo- or the epicardium.

### Potential Maps

**FIGURE 4:**
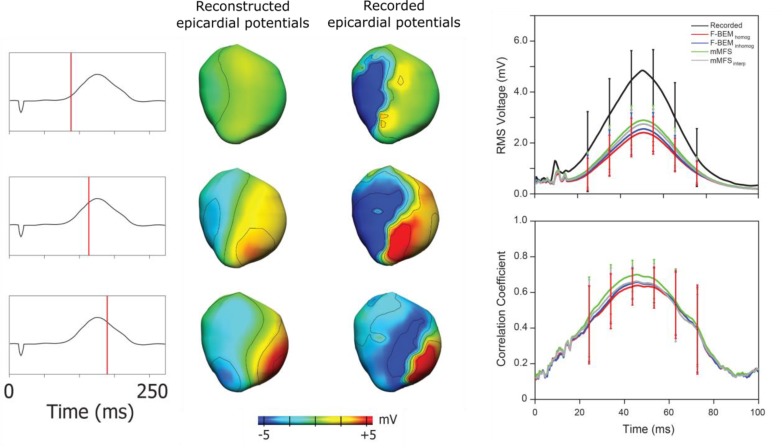
Epicardial potential maps derived from ECGI (left) and recorded (right) epicardial electrograms at three instants during ventricular activation during epicardial pacing from the anterior RV free wall (ECG shown left). During the early and late phases of activation, only small regions of the heart are activated, leading to distorted values of error metrics such as correlation (right, showing different reconstruction approaches in different colors). Adapted with permission from Bear et al. (2018b).

In addition to electrograms, potentials can be visualized spatially as potential maps that vary in time. Hence, validation is often performed using the same data set as for electrograms through global quantitative comparisons of reconstructed potential maps to the ground truth signals, using correlation and error metrics over time (Oster et al., 1997; Bear et al., 2018b). One limitation of quantitative comparison is that during the early and late phases of activation, only small regions of the heart are activated, leading to distorted values of signal-to-noise and global error metrics (see **Figure 4**). It remains unclear if specific characteristics during these time periods can be accurately reconstructed, such as the initial site of activation (based on the location of epicardial deepest negative potential) or regions of current sources (similar to ST segment elevation). However, potential maps are only beginning to be used in clinical practice (Jamil-Copley et al., 2015) and assessment of individual electrograms for signal amplitude, activation time or specific characteristics is currently more clinically practical.

### Activation/Recovery Maps

Activation and recovery time maps provide very useful static isochrone images of the cardiac electrical depolarization/repolarization sequence. These isochrones may be used clinically to identify several quantities: (a) sites of initiation of activation by focal arrhythmias, e.g., premature ventricular contraction (PVC) origin; (b) delineation of reentry circuits, e.g., macroreentrant atrial flutter (Shah et al., 1997), or (c) abnormalities in propagation, such as areas with delayed activation (Irie et al., 2015), or those with slow or abnormal conduction, e.g., scar-related ventricular tachycardia substrate, or (d) areas with recovery abnormalities, e.g., large recovery gradients (Vijayakumar et al., 2014; Leong et al., 2017). To date, the majority of validation studies have concentrated on the accuracy of activation mapping (Oster et al., 1997; Zhang et al., 2005; Han et al., 2011; Oosterhoff et al., 2016; Bear et al., 2018b), with only a few studies assessing recovery (Van Dam et al., 2009; Cluitmans et al., 2017). The earliest site of activation of paced beats is often reconstructed and compared to the known location of pacing, yielding a localization error. For example, animal validation studies with pacing localization exist for potential-based (Cluitmans et al., 2017; Bear et al., 2018b) and activation/recovery-based approaches (Han et al., 2011; Liu et al., 2012; Oosterhoff et al., 2016).

**FIGURE 5:**
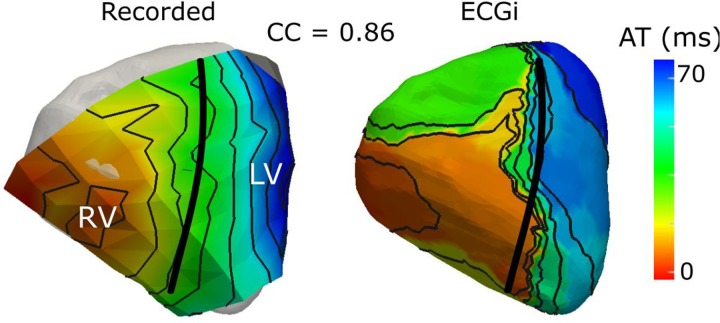
Validation of ECGI derived activation maps using an *ex vivo* torso tank set up has demonstrated that, in electrical dyssynchrony, ECGi (right) produces a “line of activation block” that is not present in recorded signals (left) using an epicardial electrode sock [adapted with permission from Bear et al. (2018a)].

Apart from beat origins, validation studies of activation- and recovery-time maps tend to assess global accuracy, providing correlation and relative error metrics. This approach may overlook other characteristics/abnormalities, such as steepness of recovery gradients, or the presence/absence of conduction block or localized changes in velocity of activation (conduction). One study (Bear et al., 2018a) has recently demonstrated that in left bundle branch block, ECGI can compress local activation to create an artifactual “line of block,” which is not reflected in correlation values (**Figure 5**).

One of the difficulties in activation/recovery map validation is the need to derive the activation/recovery times from electrograms and thus their dependence on the signal processing method used. Most applications use the gold standard method for deriving activation/recovery times from directly recorded signals (defined as the time of minimum and maximum derivatives, respectively, of the unipolar electrogram). However, these gold standard methods are typically imprecise, both for actual recordings and for ECGI-derived electrograms, and alternative methods may be required (Erem et al., 2011; Duchateau et al., 2016; Cluitmans et al., 2017). These methodology-linked variations in derivations of activation/recovery times may impact overall performance estimate.

### Phase Maps

Phase is a feature that was initially designed to decompose action potential signals, the basic concept being to discard amplitude and focus on the temporal sequence (resting state, activation, refractory period, and repolarization). Between two consecutive activations of the tissue, the phase will increase in a monotonic way from -π to +π (or from 0 to 2 π if preferred). When applied to optical mapping recordings of complex cardiac arrhythmia (atrial and VF), phase is an effective feature in analyzing spatiotemporal organization (Gray et al., 1996). The spatial representation of the instantaneous phase allows one to identify focal activity and/or local reentry using features computed directly from the spatial organization of the phase signal (phase divergence and phase singularities). Such representations have sparked clinical interest in using phase mapping on electrical recordings as a means of detecting organized stable drivers during fibrillation, and potentially target them for ablation therapy (Umapathy et al., 2010; Narayan et al., 2012; Haissaguerre et al., 2013).

**FIGURE 6:**
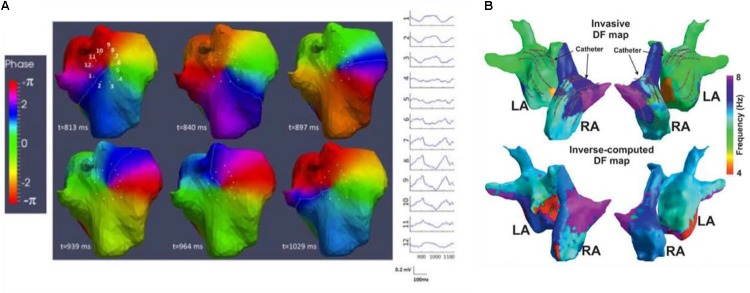
During atrial (AF) or ventricular fibrillation, the mechanism of fibrillation can be assessed using phase and/or frequency mapping. **(A)** [adapted with permission from Haissaguerre et al. (2014)], ECGI derived phase maps of ≥1000-ms-long AF window show reentry events involving the posterior upper right atrium; sites 1–12 show the pre-phase electrograms around its core. By tracking phase singularities (PS) for reentrant activity of at least one full rotation, PS density maps illustrate arrhythmogenic drivers that may be targets for ablation. **(B)** [adapted with permission from Pedrón-Torrecilla et al. (2016)], dominant frequency (DF) using invasive CARTO (top) and non-invasive ECGI (bottom) in AF patients demonstrate good correspondence. Region of high DF (purple) identify arrhythmogenic drivers that may be targets for ablation.

An efficient tool commonly used to convert time-varying signals from the voltage domain to the phase domain is the Hilbert Transform. This conversion from voltage to phase does not meet the objective of having a monotonic signal that ranges from -π to +π (or in general an interval of 2 π) between two consecutive cycles for signals with multiple deflections, such as bipolar or noisy electrograms. For this reason, electrograms are typically smoothed prior to conversion. Depending on the filtering chosen for the preprocessing step, the resulting phase maps may lead to over/under-estimation of the extent of re-entrant cycles (Rodrigo et al., 2017). The lack of methodological consensus on both the preprocessing and the definition of spatially or temporally stable phase singularities has resulted in ongoing debate over phase mapping techniques (Vijayakumar et al., 2016; Kuklik et al., 2017). To minimize such ambiguities, it is advisable to incorporate electrogram characteristics and the time-domain activation sequence into the analysis (as demonstrated in **Figure 6**).

Though applicable to the ventricles (Umapathy et al., 2010), contemporary ECGI applications are most commonly atria based, with validation using some clinical patient data (Metzner et al., 2017) and, more extensively, simulated data (Dubois et al., 2016; Figuera et al., 2016; Pedrón-Torrecilla et al., 2016; Kuklik et al., 2017; Rodrigo et al., 2017). Validation using experimental data is still needed. Phase can be validated in a similar manner to electrograms, using correlation and relative error to assess the global topology of the phase signals. However, phase signals at a particular electrode are themselves not of clinical interest, but rather the spatial distribution and temporal evolution of phase that allows the detection of divergence/singularity points. As such, in addition to correlation and relative error, we support the use of error metrics that assess localization accuracy of the divergence/singularity points or derived maps, such as the weighted under-estimation or the weighted over-estimation indicators (Figuera et al., 2016). Another useful approach entails computing the Phase Locking Value (PLV), which estimates the synchronicity between two signals, without regard to the constant phase shift between them (Dubois et al., 2016).

### Dominant Frequency

In addition to phase, frequency is often used in the analysis of atrial and VF, where localized sites of high (or dominant) frequency (DF) serve as target ablation sites to eliminate AF (Sanders et al., 2005) (as seen in **Figure 6**). Because the conventional computation of frequency is done over a time segment, DF produces time-averaged frequency information over space. An absence in stability of the arrhythmia can lead to errors in DF estimation. Metrics to assess stability can be computed, such as the regularity (Everett et al., 2001; Sanders et al., 2005; Sánchez et al., 2017), organization, or coupling indices (Faes and Ravelli, 2007) and they indicate whether the activation is regular/periodic. Sequential analysis can be used to track the movement and determine the stability of DF sites over time (Salinet et al., 2014). In addition, the presence of shape-related and not only rhythm-related DF components can result in incorrect identification of the activation rates in the atria. As with phase, studies have reported controversial results, likely due to the different approaches for signal processing, DF computation, and interpretation. We refer readers to a review by Ng et al. (2007). That explores the technical requirements and limitations of dominant frequency analysis of AF signals.

Like phase mapping, current technical validation of ECGI derived dominant frequency has been limited to the atria and to the use of endocardial mapping measurements (Pedrón-Torrecilla et al., 2016; Zhou et al., 2016) and simulations (Figuera et al., 2016; Rodrigo et al., 2017) to provide ground truth data. Validation of frequency-based estimates can be performed using relative or absolute error, correlation to assess the global map distribution, and, more importantly, error metrics to assess localization of the region of dominant frequency, e.g., direction of DF gradient, either from left atrium to the right atrium, or weighted under/over-estimator indicators (Figuera et al., 2016), as well as other metrics that may be derived such as regularity/organization indexes.

## Pathological Validation

The major goal of ECGI is to be useful as a clinical tool to aid in diagnosis and therapy planning of specific pathologies and/or arrhythmias by identifying the mechanisms underlying these electrical disorders and their response to therapy. As such, validation of ECGI to extract the information applicable to a specific pathology/arrhythmia is essential. As mentioned previously, the quantitative accuracy of the reconstructed quantities may not be as important as their ability to separate healthy and diseased states. However, ECGI needs to be accurate enough to ensure that conclusions drawn from specific features are not actually a misinterpretation of artifacts in the solution. Moreover, clinicians typically want insight into the characteristics of a pathology and not just a binary classification. Such depth and detail are particularly important because conclusions drawn from ECGI can lead to the identification and implementation of costly, and potentially life-saving, treatment plans, such as guiding ablation therapy or implanting ICD or CRT devices.

Here we identify some pathologies and/or arrhythmias for which ECGI may be applicable, along with associated features desired for clinical applications. For a summary of the results from previous ECGI studies of arrhythmogenic substrates, we refer the reader to the review by Rudy (2013).

### Atrial Arrhythmias

One of the first extensively clinically explored applications of ECGI was to identify the specific mechanisms of arrhythmia onset and/or maintenance for ablation therapy in atrial tachycardia (AT) and fibrillation (AF). ECGI is currently used to determine the specific patterns underlying activation during arrhythmia (i.e., focal vs. re-entrant) through phase mapping (Cuculich et al., 2010; Roten et al., 2012) and to localize the drivers of this activation using either the estimated dominant frequency (Pedrón-Torrecilla et al., 2016), and/or the divergence/singularity points through phase mapping (Haissaguerre et al., 2013, 2014). These drivers have been found to be correlated to the ablated regions in patients with successful outcome (Haissaguerre et al., 2013; Zhou et al., 2016) and are now being targeted directly in clinical validation studies (Haissaguerre et al., 2014; Knecht et al., 2017). While these studies indicate these drivers are targeting the arrhythmogenic substrates, one cannot rule out the possibility of false-positive errors. Technical validation has demonstrated that coarse patterns of DF (seen in **Figure 6**), singularity points, rotor/focal source density, etc., can all be accurately localized with ECGI (Modre et al., 2003; Haissaguerre et al., 2014; Dubois et al., 2016; Figuera et al., 2016; Metzner et al., 2017), but the specific ECGI features that most effectively localize the arrhythmogenic substrate(s) in AT/AF are still under debate. Pathological validation of this kind is nearly impossible as often multiple ablations are performed during a procedure, and it is unclear if all, some, or only the last locations identified the true arrhythmogenic substrate(s). As such, indirect clinical validation studies that show improved patient outcome or reduced procedural times are currently the best option.

### Arrhythmogenic Substrate Identification for Ventricular Arrhythmias

As with atrial arrhythmias, one of the primary goals of ECGI is the identification and understanding of arrhythmogenic substrates for the onset and maintenance of ventricular tachycardia (VT) and fibrillation (VF) to aid in treatment planning, e.g., to guide pharmacologic therapy, ICD implantation, or catheter ablation. Below we outline the major areas ECGI has been used for substrate identification in VT and/or VF.

#### Premature Ventricular Contraction

**FIGURE 7:**
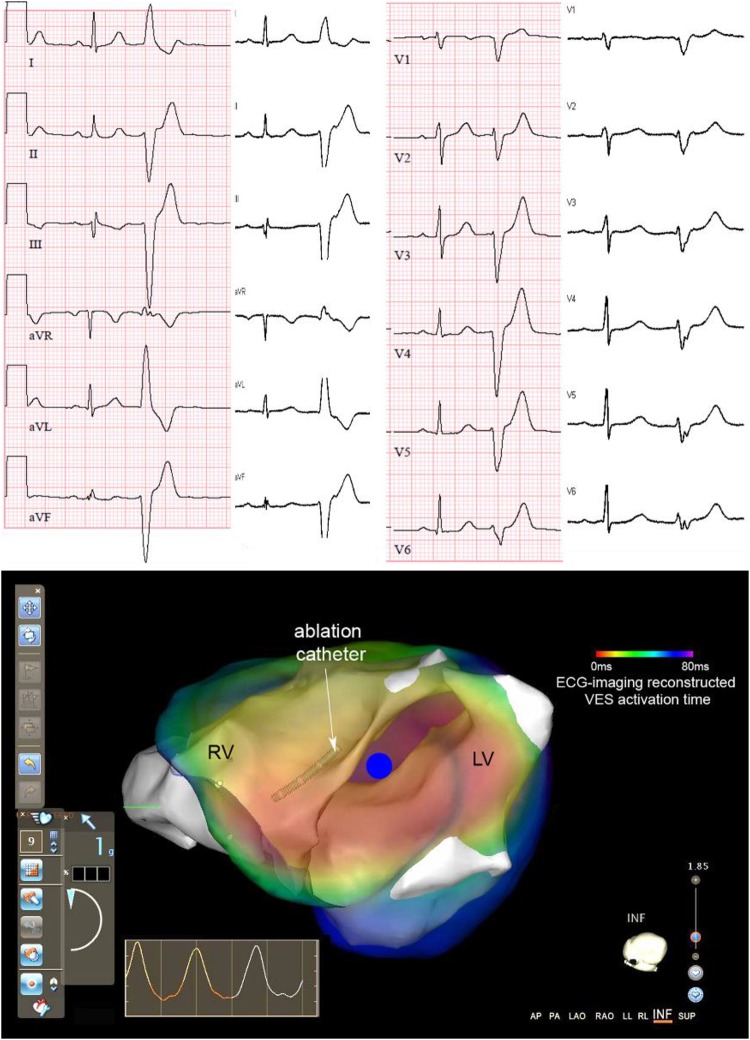
Example of ECGI-guided ablation in a patient. Top: 12-lead ECG recorded during ECGI (white background) and clinical recording during symptoms (pink background), both with a sinus beat and subsequent premature ventricular complex (PVC). Bottom: Live view as visible to the cardiologist-electrophysiologist during the ablation procedure when using the Carto 3 cardiac mapping system. The 3D anatomy as determined by the Carto catheter (white structures) is overlaid with the activation map of the PVC as created pre-procedurally with ECGI (red-to-blue: early-to-late activation time; blue dot: PVC origin). The cardiologist can use this live view during the procedure to navigate the catheter to the area suspected of the PVC origin. Adapted with permission from: Cluitmans et al. (2016).

Patients with isolated and monomorphic PVCs or focal VT can be treated by ablating the focal source of these arrhythmias, which typically involves long, invasive electrophysiological mapping to localize these ectopic activities. By non-invasively localizing the arrhythmogenic site or at least estimating a region of interest prior to the catheterization procedure, ECGI can provide a useful presurgical guideline for the operator, and substantially improve the planning of the procedure and thus reduce procedure times (Potyagaylo et al., 2013), an example of such guidance is demonstrated in **Figure 7**. Focal idiopathic VTs and PVCs in the absence of structural heart disease are one of the most well validated pathologies for ECGI application, as the arrhythmogenic substrate is clearly defined and one can define accuracy using a simple localization error (Schulze, 2015). For example, a commercial implementation of ECGI (ECVUE, CardioInsight, Cleveland, OH, United States) has been used to localize PVCs (Erkapic and Neumann, 2015). Their ECGI implementation localized the ventricle of origin (LV vs. RV) correctly in 95.2% of the cases, compared to only 76.6% with 12-lead ECG. Sub-localization within the ventricles was accurate in 95.2% of the cases with ECGI, compared to 38.1% with 12-lead ECG. Ablation success was similar in both groups, both acutely and at 3-month follow up. Failure of this ECGI implementation (which uses an epicardial surface source model) to localize PVCs occurred mainly in sites near the septum, especially at the LVOT or RVOT (Erkapic and Neumann, 2015). In a study using NEEES (EP Solutions SA, Yverdon-les-Bains, Switzerland), PVCs were localized in 20 patients; in 86% of the cases, the correct ventricular segment was diagnosed (Wissner et al., 2017). In a preliminary study with nine participants, the Vivo 12-lead ECGI system was able to successfully localize PVCs (defined as location of successful ablation) in 86% of the cases, whereas human-based 12-lead ECG localization of PVC had a 27% accuracy (Gordon et al., 2014).

Future validation would ideally compare old and new methods to these previous works, potentially using a common and broad data set to prevent bias. An international working group called the Consortium for ECG Imaging (CEI^1^) has recently emerged that seeks to enhance progress through collaboration and shared data repositories [EDGAR (Aras et al., 2015)], with first results very recently published (Svehlikova et al., 2018).

#### Scar Related Ventricular Tachycardia Mapping

Sustained reentrant VT (and VT-induced VF) is often formed by narrow channels of surviving tissue inside a myocardial scar. Catheter ablation is an effective treatment for scar-related VT that modifies the scar substrate, for example by destroying the tissue site at which the VT circuit exits the scar (exit sites) [ESC Guidelines Management Ventr Arrh/AHA 2017 VA/SCD guideline (Reddy et al., 2007; Kuck et al., 2010; Sapp et al., 2016)]. These scar substrates are currently identified by catheter mapping, either during artificially induced VTs (by programmed electrical stimulation), in which the morphology and critical components of the VT circuit can be delineated, or during native rhythm where the region of myocardial scar and potential critical sites can be identified from the characteristics of the measured electrograms (although less specific than VT mapping). However, these procedures can be hindered by a lack of inducible arrhythmia or, more frequently, by the presence of multiple inducible arrhythmias and the patients’ hemodynamic intolerance to the induced VTs. Additionally, native-rhythm substrate mapping involves invasive procedures with access often limited to the left endocardium. ECGI, due to its non-invasive nature, has the potential to augment catheter mapping in both enabling instantaneous reconstruction of a single PVC or a brief episode of VT and thus to provide non-invasive identification of scar substrates during native rhythm. For VT mapping, a small number of human studies have investigated the use of ECGI to map the exit sites and activation pattern of reentry circuits, using both epicardial ECGI (Wang et al., 2011; Sapp et al., 2012; Zhang et al., 2012) and more recently using epicardial-endocardial and 3D ECGI (Tsyganov et al., 2017; Wang et al., 2018). For substrate mapping, while the use of ECGI to delineate myocardial scar has been validated using MRI or voltage mapping (Cuculich et al., 2011; Wang et al., 2013; Horáček et al., 2015), studies are just emerging to examine its ability to reveal local abnormal electrograms, such as fractionated electrograms, that are suggestive of potential central pathways forming the VT circuit (Wang et al., 2018). In all of these studies, a significant challenge arises from the difficulty to establish the clinical ground truth for the exit sites and central pathway for VT; as a result, most existing validation studies are limited to qualitative or semi-quantitative evaluations. Validation studies should focus on the ability of ECGI to localize the VT exit site, using a similar metric to validations based on PVCs, as well as other critical sites, such as central pathways within myocardial scar.

#### Ventricular Fibrillation

Instantaneous whole-ventricle mapping systems such as ECGI open the possibility to also investigate VF mechanisms, by looking at focal centers or anchors of VF activity (similar to AF). Results from this field are only just emerging (Frontera et al., 2018) and could be validated with approaches that are similar to those applied in the AF field, including answering the question of whether these key spots within reentrant loops are really useful targets for ablation.

#### Wolf-Parkinson White Syndrome

Wolff-Parkinson-White (WPW) syndrome involves a long-standing altered activation sequence, different from normal sinus rhythm, as a result of abnormal conductive cardiac tissue between the atria and the ventricles that provides a pathway for a reentrant tachycardia circuit. Similar to focal VT and PVCs, localization of WPW is a natural application of for ECGI as a means to guide catheter ablation. However, the high rate of success in WPW using conventional approaches (Fitzpatrick et al., 1994) and relatively minor potential for incremental clinical benefit has limited motivation to a few studies (Berger et al., 2006; Ghosh et al., 2008).

### Risk Stratification for Ventricular Arrhythmias

In addition to identifying the arrhythmogenic substrate for ventricular arrhythmias, ECGI has more recently been proposed as a tool for risk stratification for arrhythmia, particularly VF and sudden cardiac death. By imaging and quantifying the important substrates for arrhythmia such as conduction and repolarization abnormalities, ECGI may be used for screening of the general public to identify those at risk. Initial studies have begun to identify these potential arrhythmogenic substrates, such as the presence of slow discontinuous conduction and steep dispersion of repolarization in the RVOT of Brugada patients (Zhang et al., 2015), the presence of repolarization abnormalities in ARVC patients (Andrews et al., 2017), steepness of recovery gradients (Vijayakumar et al., 2014), and recovery substrates in sudden cardiac death survivors (Leong et al., 2017). However, much work is still needed in this area not only to confirm these substrates but also to develop and validate risk stratification markers that identify them and to demonstrate clinical utility.

### Heart Failure and Cardiac Resynchronization Therapy

Cardiac Resynchronization Therapy (CRT) through biventricular pacing can improve outcomes in patients with heart failure and with low ejection fraction. However, the effect varies widely with approximately a third of patients failing to respond. To improve CRT response rates, recommendations emphasize attention to low resolution electrical parameters from the 12-lead ECG (i.e., left bundle branch block and QRS duration ≥150 ms). However, detailed understanding of the spatiotemporal excitation pattern in the failing human heart and its relationship to a CRT response are lacking. ECGI has been used to fill some of this gap, providing new insights into the electrical substrates specific to responders (Varma et al., 2007; Berger et al., 2011; Ghosh et al., 2011; Ploux et al., 2013), including abnormal activation patterns with slow conduction and/or line of block and electrical dyssynchrony, both within and between the ventricles. With knowledge of these substrates, new ECGI-derived criteria have been developed, demonstrating improved responder selection compared to the current standard metrics (Ploux et al., 2013). Clinical validation of these metrics in the form of large-scale multi-center trials is still required to demonstrate that these metrics are capturing the correct electrical substrates. Technical validation has demonstrated that the presence of functional lines of block seen clinically in these patients may in fact be an artifact of ECGI (Bear et al., 2018a) (see **Figure 4**). Despite this, ECGI has reliably and accurately detected electrical dyssynchrony, resynchronization by biventricular pacing, and the site of latest activation, providing more information than the 12-lead ECG. Further validation of ECGI methods in the presence of heart failure and/or electrical dyssynchrony could improve reconstructions to remove these artifacts and increase the sensitivity and specificity of these markers, and possibly to develop methods for targeted left ventricular lead placement.

## Clinical (And Socioeconomic) Validation

Electrocardiographic imaging validation studies in the past have typically focused on the technical or pathological validation and until recently, large-scale studies using ECGI clinically have not been possible. With the commercialization of ECGI systems, their use on a large scale in hospitals worldwide is now a possibility. As such, this section will deal with the need for studies to address the actual clinical benefit in terms of personalized understanding of disease mechanisms and its potential to open new avenues for therapy, improved patient outcome, and improved workflow. As far as we know, the following systems are currently in development for commercial purposes: the CardioInsight^TM^ Noninvasive 3D Mapping System (Medtronic), the Non-Invasive Electrophysiological Mapping (NIEM) system (EP Solutions SA), and the View Into Ventricular Onset (Vivo) system (Peacs, Catheter Precision).

### Clinical Application Domains

The potential applications of ECGI (and their validation) fall into three major categories: (1) Personalized disease understanding and diagnosis; (2) Therapy guidance; and (3) Enabling innovations.

#### Personalized Disease Understanding

Many of the studies discussed in section “Pathological Validation” have investigated disease mechanisms with ECGI and proposed parameters that carry relevant clinical information. For example, ECGI has been used to uncover repolarization gradients that are more pronounced in symptomatic than asymptomatic LQTS patients (Vijayakumar et al., 2014). Similarly, the more detailed information provided by ECGI has demonstrated the presence of larger ventricular electrical dyssynchrony in CRT responders providing improved CRT patient selection compared to standard 12-lead ECG (Varma et al., 2007; Ploux et al., 2013). These retrospective studies are important to help understand the underlying disease mechanisms and define the parameters or markers that may guide therapy. But to fully demonstrate that this information carries value for an individual patient and could guide therapy or patient selection requires further investigations using large-scale prospective studies.

### Therapy Guidance

Most clinical studies have aimed at using ECGI for therapy guidance. To date the focus has been on demonstrating a decrease in radiofrequency ablation and procedural times for atrial arrhythmia substrates or PVC origin ablation therapy, as pathological studies, discussed in sections “Atrial Arrhythmias” and “Premature Ventricular Contraction,” have shown that accurate localization of these substrates is feasible. A clinical validation study for AF has reported that using ECGI to identify ablation targets with phase mapping, as seen in **Figure 6**, significantly reduced radio-frequency delivery (28 ± 17 vs. 65 ± 33 min) and thus procedure times for successful outcome, compared to standard methods (Haissaguerre et al., 2014). Likewise, for PVC localization, clinical validation has shown the number of radiofrequency energy applications was significantly lower in the group with CardioInsight-based localization (two applications) in comparison with the control group (four applications) and procedure times were shorter (Erkapic and Neumann, 2015).

In order to be used in other pathologies, it is necessary to overcome technical challenges that currently limit the applicability of ECGI during invasive cardiac procedures (e.g., the need to integrate defibrillation patches and 3-dimensional mapping systems). ECGI might then guide therapy by characterizing electrical substrate(s) in more detail than is possible from the 12-lead ECG. For example, ECGI provides detailed electrical activation patterns of the left (LV) and right ventricle (RV), which could guide CRT implantation by suggesting the optimal placement of the LV lead (Ploux et al., 2014; Bear et al., 2018a). ECGI may also enable risk stratification and guide ICD implantation for sudden cardiac death by imaging of activation and recovery abnormalities, both important arrhythmogenic substrates (Vijayakumar et al., 2014).

For all of these applications, prospective clinical trials are also needed to validate the clinical benefit. ECGI for AF is leading the way, with one such multi-center trial currently underway (AFACART^2^) aiming to evaluate the effectiveness of ECGI for persistent AF mapping and ablation procedures in comparison to conventional methods. Initial results recently published from this trial have demonstrated a persistent AF termination success of 72% (*n* = 118), with no significant difference among the 8 European centers in the study (Knecht et al., 2017).

### Enabling Innovative Therapies

Electrocardiographic imaging carries the potential to permit new and innovative therapies. A recent study has demonstrated the feasibility of combining ECGI with non-invasive delivery of ablative radiation, suggesting that clinicians may be able to completely non-invasively target VT substrate with radiotherapy (Cuculich et al., 2017). Accurate identification and localization of arrhythmogenic myocardial substrate could facilitate further progress in the development of safe, effective therapeutic interventions.

Furthermore, studies are currently investigating the used of ECGI with tagged MRI/CT or speckle-tracking echocardiography (Dawoud et al., 2016). Combining non-invasive imaging of electrical and mechanical function and their interaction in situ could provide new insights that would be extremely valuable for characterization of disease mechanisms and development of treatment strategies. The wealth of information provided by ECGI, and its non-invasive nature, might open the door to more novel innovations.

### Demonstration of Added Clinical Value

#### Improved Patient Outcome

One obvious goal of clinical validation studies is to show that ECGI can improve patient outcome. This requires long-term, large-scale (thus expensive) clinical trials to validate the findings arising from small scale technical or pathological validation studies; one such example is the AFACART study. These types of studies are needed to prove patient benefit in terms of reduced recurrence rates after such ECGI-guided procedures as PVC ablation (Erkapic and Neumann, 2015) and AF ablation (Haissaguerre et al., 2014) or improved CRT patient selection or lead positioning (Ploux et al., 2013). There are many potential reasons why ECGI may not improve patient outcome including: (1) a lack of understanding of disease mechanisms, making it difficult to specify how ECGI could prove useful (e.g., rotors in AF or lack of response in CRT); (2) the lack of clinical need (PVC ablation can often be performed without expensive or complex mapping systems); and (3) the preliminary nature of many studies seeking to show ECGI results prior to randomized control trials. Large, multi-center studies are currently being designed or are underway to investigate the benefit of ECGI for various diagnostic and therapeutic purposes.

#### Improved Workflow and Reduced Cost

Another improvement that could be provided by ECGI is in workflow and (socio)economic cost. Such benefits are particularly important if ECGI is to be considered as a tool for risk stratification and general population screening. The associated validation studies should focus on finding the optimal balance between time and financial investment (ECGI is more expensive and more time-consuming than a standard ECG) vs. benefit for society. These benefits could include: lower hospitalization rates with AF and VT ablation, shorter procedural times (or lower variability of procedure results, making hospital planning more reliable), socioeconomic benefit due to improved patient selection for ICD therapy, etc. Commercial implementations of ECGI are essential for this and preferably include regulatory approval and financial reimbursement. To our knowledge, Medtronic’s CardioInsight system is the only FDA-approved ECGI implementation, and no ECGI implementation is currently being reimbursed by health care insurers.

### Clinical Adoption

Clinical adoption of ECGI depends critically on technical, pathological and clinical validation, but also on practicability, demonstration of cost-effectiveness, and appropriate reimbursement mechanisms. Additionally, if levels of confidence or uncertainty were reported to clinicians for ECGI reconstructions in individual cases (taking into account levels of noise, defective electrodes, regions that are hard to reconstruct, etc.) it might further improve clinical reliance on ECGI results. In general it is not yet clear what is the best context to apply ECGI to improve health care, and it is important to realize that while such benefits might lie in improved diagnosis, therapy guidance, or patient outcome, they may also result from improved workflow (reduction of procedural times, less variability in procedure duration) and reduced cost (shorter or fewer hospitalizations). Whatever the focus, such studies will cost time and money and will benefit from focused clinical applications.

## Consensus on Designing a Validation Study

In designing and presenting a validation study, there are many choices that can have a large impact of the validity of the results. In this section, we provide a consensus on the “best” approaches. This consensus is based on discussions among the authors at recent conferences (Computing in Cardiology [CinC] 2015–2017; Frontiers in Computational Electrocardiology [FiCE] 2016; and the International Society for Computerized Electrocardiology [ISCE] conference 2015), and during other meetings.

### Data Selection

Data sets come in various forms: simulated, experimental (*ex vivo* torso tank or *in vivo* animal model), and clinical as summarized in Macleod and Brooks (2000). The ideal validation would include data in more than one form (i.e., simulated and experimental), and include multiple sources (i.e., multi-institutional clinical and/or experimental data). Depending on a single data set can lead to algorithms becoming biased or tuned to these data and ultimately failing on other independent data. Validation using *in vivo* animal and clinical models are particularly needed to address the technical questions surrounding accuracy, feasibility, and reliability. Using data from multiple sources/forms adds to the rigor, reliability, and reproducibility of the results.

However, obtaining such data sets is often impossible for individual labs due to the large costs and/or lack of available facilities. Collaborative efforts have and will continue to help overcome this problem. To enable such projects, an international consortium has created an open platform for sharing data, which is populated with datasets (14 datasets from 8 different centers as of the submission of this manuscript). This database is called the Experimental Data and Geometric Analysis Repository (EDGAR^3^) and is hosted at the University of Utah, as described in Aras et al. (2015). EDGAR has already supported a number of cross-laboratory validation studies published in both journals and relevant conferences (Chamorro-Servent et al., 2016; Schulze et al., 2017; Cluitmans et al., 2018; Svehlikova et al., 2018).

An essential requirement for the data sets of choice is to define an appropriate ground truth, a goal that as described above is not always easy with experimental or clinical data. We recommend including the error of experimentally determined “ground truth” whenever possible, to give context to validation studies, e.g., the accuracy in localization of an ablation site.

### Technical, Pathological or Clinical Validation

Despite the distinction between technical, pathological, and clinical validation in this review, in practice validation should be seen more as existing on a continuum (see **Figure 1**). In the early days, validation studies of a purely technical nature were common, but with time, studies have included pathological aspects; however, there are still only few published purely clinical/socioeconomical validation studies. Given the increased application of ECGI for clinical use, future validation studies should contain aspects of at least two forms of validation, if not all three, by giving a clinically oriented perspective to results. For example, validation of a new formulation for ECGI in a purely technical fashion might report that electrograms are now reconstructed with a correlation slightly better than previously reported methods. However, putting this result into perspective in terms of quantifying, say, improved accuracy to detect arrhythmogenic substrate for ablation targeting, and/or improved patient outcome, would provide for a substantially more powerful contribution.

### Metrics for Validation

To assess ECGI accuracy in technical validation studies, a variety of metrics have been derived and reported. Each metric comes with its own advantages and disadvantages, supporting the use of several different metrics to carry out a useful evaluation. Here, we discuss these metrics, their current uses and their limitations.

#### Global Comparisons

In general, it is important to quantify accuracy in both time and space in a global sense; to quantify similarity in shape (e.g., temporal waveforms or spatial potential/activation distributions), and amplitude (e.g., voltage). Metrics to quantify global accuracy include:

• Correlation: this metric is often used in ECGI validation as it quantifies the accuracy in shape regardless of any amplitude inaccuracies. However, it may not be clear to clinicians what the different values of correlation correspond to in terms of diagnostic or therapeutic accuracy. For example, electrode 5 in **Figure 3** shows a high correlation coefficient that does not reflect the fact that the reconstructed electrogram misses the (clinically informative) initial positive deflection shown by the ground truth.
• Phase Locking Value (PLV): PLV estimates the average synchronicity between two signals, without regard to the constant phase shift between them (Dubois et al., 2016). This is useful in the evaluation of phase, where the exact synchronicity of the signals is less important.
• Relative or Absolute Error: these metrics are used to quantify the accuracy in amplitude (or timing in the case of activation maps). Relative error has the advantage of defining the accuracy relative to the size of the feature being measured, meaning it can be more clear whether an absolute difference is very significant or not (e.g., an absolute error of 2 mV is not very important when the gold standard is 25 mV, but more substantial when it is 4 mV). However, like correlation, relative error is less meaningful to clinicians. Furthermore, relying on relative error can badly miss critical similarities, e.g., a slight time misalignment of activation could result in a large value of squared error. Conversely, small abnormalities (e.g., a small infarcted region or scar) that are missed in a reconstruction may not significantly affect global relative error.

#### Local Comparisons

As discussed previously, correlation and/or relative/absolute error correspond to accuracy on a global scale (**Figures 3**–**5**), but neglect the accuracy of the finer, potentially more important, characteristics. The following metrics have been developed to quantify the accuracy of specific characteristics.

• Localization Error: this metric is used to define the accuracy in localization of specific features (e.g., in space: site of earliest activation; or in time: timing of first breakthrough). While it is easy to interpret, it is not always the most clinically relevant metric. For example, a 5 mm error in PVC localization is more significant than a much larger error in distance, if the location is incorrectly identified as the LV instead of the RV compared to when both correct and incorrect sites are in the same chamber. Some authors have quantified localization to a predefined segment of the heart [e.g., segmentation according to the AHA classification (Austen et al., 1975)] but these indicators can also be misleading, for example if the correct location lies just on one side of a segment boundary and the reconstructed location is nearby but just on the other (Sapp et al., 2017). Moreover, estimation of activation time and site of first activation from reconstructed electrograms is itself a difficult problem that is currently the topic of active investigation by many groups (Erem et al., 2011; Duchateau et al., 2016), including the CEI workgroup on Activation and Recovery times^4^.
• Weighted under/over-estimation indicators (Figuera et al., 2016): are used to assess the localization accuracy of larger features such as the divergence/singularity points maps or ischemic region detection. They define the percentage of misjudged region (either positive or negative), by the ECGI reconstruction. These indicators are highly dependent on the size of the region being assessed, and like relative error can miss critical similarities if the region reconstructed is only slightly misaligned.

While each metric has its merits, the limitations mean it is advisable to use all of these metrics with caution and ideally none should be the only metric used in a technical validation study. That is, for each feature, other clinically relevant, ideally quantitative, comparisons are necessary. These should be based on the characteristics of each feature one wants to reconstruct, e.g., electrogram fractionation, ST-segment elevation, DF or phase singularity points, etc.

Given the sensitivity of inverse reconstructions to noise and model error (which could well be from physiological causes such as respiratory or contractile motion), studies should report the distribution of cumulative results across an ensemble of beats, and not just a single case or a mean reconstruction. For example, box plots showing the median, the quartiles and outliers can be a better choice. More broadly, new metrics will likely need to continue to be developed to achieve these goals.

### Reproducibility

A thorough description of all experiments and analyses is essential to allow for reproducibility and reliable comparison to other methods. Such descriptions include the full workflow, from scanner settings to segmentation to therapy application to metric calculation. Clinical study design should be fixed before being executed and registered publicly (e.g., ClinicalTrials.gov). The investigators may also choose to share the data and/or methods used (e.g., via collaboration or the EDGAR database), allowing other research groups to compare approaches and improve accuracy.

### Statistics

The statistical analysis performed for any validation study depends on the form of validation, comparisons made, data set used, and many other factors. The choice of statistical test must be made with care. Unfortunately, many studies use a simple *t*-test, missing potentially important information that may be obtained by taking into account other dependent and independent factors that can influence accuracy (e.g., subject, cardiac sequence, or region of the heart for reconstruction), as well as by including comparisons based on carefully designed metrics of effect size as well as on more comprehensive distributional comparisons. Incorporating these factors into the analysis may help reveal the source of outliers, define regional accuracy, or provide an indication of confidence in the results. In particular, including effect size in addition to the more traditional mean or median-based statistics allows the evaluation of the clinical relevance of statistically significant differences, i.e., outcomes may fall in statistically significantly different groups, based on means or median, but overlap of distribution between those groups may prevent reliable prediction on a per-patient level.

## The Future of Ecgi

Electrocardiographic imaging has a rich history, from the first model development and animal studies in the 1970s, through extensive technical validation studies in computer simulations, *ex vivo* torso tank and *in vivo* animal studies, to its more recent human use to understand pathological mechanisms of diseases and guide clinical procedures in patients. Continued technical, pathological, and especially clinical validation will be essential for full adoption of ECGI as clinical technique. We have highlighted the accomplishments of the last decades, and have given pointers to what remains to be done for specific diseases and applications. Specifically, showing *clinical benefit* is essential for the adoption of this powerful technique. Such benefit may lie in patient outcome improvement, workflow improvement, or cost reduction. Future studies should focus on these aspects to achieve broad adoption of ECGI, but only after the technical challenges have been solved for that specific application/pathology. Importantly, one should realize that ECGI remains a tool used for one half of patient treatment, with the other half being the therapy given. If the therapy itself is inadequate or incomplete, it should be no surprise that the application of ECGI will not directly improve procedure outcome. Similarly, if disease mechanisms are not completely understood, the therapeutic value of ECGI-detected substrate is limited. Continued interaction between engineers, basic scientists and physicians remains essential to find a hybrid between technical achievements, pathological mechanisms insights, and clinical benefit.

## Author Contributions

MC and LB conceived of the presented idea. All authors discussed and contributed to the writing of the final manuscript.

## Conflict of Interest Statement

MC is employed by Phillips Research. PvD is the cofounder/owner of Peacs BV. OD is on the scientific advisory board of EP solutions. JS has received research support, consultant reimbursement or honoraria from from Biosense Webster, Medtronic and Abbott. The remaining authors declare that the research was conducted in the absence of any commercial or financial relationships that could be construed as a potential conflict of interest.

## Footnotes

1 http://www.ecg-imaging.org/workgroups/pvc-localization

2 https://clinicaltrials.gov/ct2/show/NCT02113761

3 http://edgar.sci.utah.edu/

4 http://www.ecg-imaging.org/workgroups/activation-recovery-times-detection
